# P-99. Comparative Analysis of False Positive Results in Low and High Risk U.S. Populations: New Access HIV Ag/Ab combo Assay vs. Abbott ARCHITECT HIV Ag/Ab Combo Assay

**DOI:** 10.1093/ofid/ofae631.306

**Published:** 2025-01-29

**Authors:** Danielle L Maher, Saeed Jortani, Fred S Apple, Robert H Christenson

**Affiliations:** Beckman Coulter Inc, Edina, Minnesota; University of Louisville, Louisville, Kentucky; Hennepin Healthcare/HCMC & University of Minneotsa, Minneapolis, Minnesota; University of Maryland School of Medicine, Baltimore, Maryland, USA, Maryland

## Abstract

**Background:**

The fully automated 4^th^ gen HIV Ag/Ab combo assay by Beckman Coulter, Inc. on the DxI 9000 Access Immunoassay analyzer allows for separate reporting of HIV-1 p24 Ag and HIV-1/HIV-2 Ab results, with a time to first result of ~30 minutes and a sample volume of 60µL. To evaluate its performance, a U.S. multisite study was conducted to compare the Access HIV Ag/Ab combo assay with the ARCHITECT HIV Ag/Ab Combo assay. False positive rates were assessed in low and high risk U.S. populations with unknown HIV status by comparing the new Access HIV Ag/Ab combo assay with the ARCHITECT HIV Ag/Ab Combo assay.1

Footnotes

**Figure:**
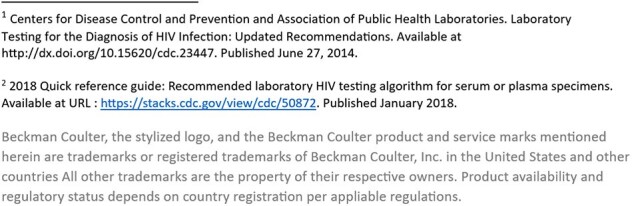

**Methods:**

The evaluation tested 6,981 low risk and 799 high risk samples (adult, pregnant, pediatric) at 3 clinical trial sites. Following the CDC recommended algorithm, samples that initially tested reactive were subjected to duplicate testing, if repeatedly reactive, were further confirmed using the HIV 1/2 differentiation assay (Geenius HIV-1/2 Supplemental Assay, Bio-Rad), and the HIV-1 RNA PCR Assay (Aptima HIV-1 Quantitative Dx Assay, Hologic, Inc).2

**Results:**

The Access HIV Ag/Ab combo assay showed a lower incidence of false positive results (n=25) in the low risk group with a specificity of 99.6% (n=6981; 95% CI: 99.5-99.8%) compared to the ARCHITECT HIV Ag/Ab Combo assay (n=87) with a specificity of 98.8% (n=6981; 95% CI: 98.5-99.0%). Similarly, in the high risk group, the Access HIV Ag/Ab combo assay yielded fewer false positive results (n=1) with a specificity of 99.9% (n=799; 95% CI: 99.3-100.0%) than the ARCHITECT HIV Ag/Ab Combo assay (n=11) with a specificity of 98.6% (n=799; 95% CI: 97.6-99.2%).

**Conclusion:**

Falsely reactive HIV test results can have significant consequences for patients and healthcare providers. Selecting an appropriate HIV assay with high sensitivity and specificity is essential. This study provides evidence that the Access HIV Ag/Ab combo assay is an attractive alternative to the ARCHITECT HIV Ag/Ab Combo assay with fewer false positive results, suggesting improved accuracy in identifying HIV infections in both low and high risk U.S. populations.

**Disclosures:**

**Fred S. Apple, PhD**, Mindray: Advisor/Consultant|Werfen: Advisor/Consultant **Robert H. Christenson, PhD, DABCC, FADLM, FACC**, Beckman Coulter: Advisor/Consultant|Beckman Coulter: Grant/Research Support|Beckman Coulter: Honoraria|Becton Dickinson: Advisor/Consultant|Becton Dickinson: Grant/Research Support|Becton Dickinson: Honoraria|QuidelOrtho: Advisor/Consultant|QuidelOrtho: Grant/Research Support|QuidelOrtho: Honoraria|Roche Diagnostics: Advisor/Consultant|Roche Diagnostics: Board Member|Roche Diagnostics: Grant/Research Support|Roche Diagnostics: Honoraria|Siemens Healthineers: Advisor/Consultant|Siemens Healthineers: Board Member|Siemens Healthineers: Grant/Research Support|Siemens Healthineers: Honoraria

